# Unicompartmental knee arthroplasty in patients aged less than 65: Combined data from the Australian and Swedish Knee Registries

**DOI:** 10.3109/17453671003587150

**Published:** 2010-03-31

**Authors:** Annette W-Dahl, Otto Robertsson, Lars Lidgren, Lisa Miller, David Davidson, Stephen Graves

**Affiliations:** ^1^Department of Orthopaedics, Clinical Sciences Lund, Lund University HospitalSweden; ^2^Data Management and Analysis Centre, Discipline of Public Health, University of AdelaideAustralia; ^3^The Australian Orthopaedic Association National Joint Replacement Registry

## Abstract

**Introduction and purpose:**

In recent years, there has been renewed interest in using unicompartmental knee arthroplasty (UKA). Several studies have reported increasing numbers of UKAs for osteoarthritis in patients who are less than 65 years of age, with low revision rates. To describe and compare the use and outcome of UKA in this age group, we have combined data from the Australian and Swedish knee registries.

**Patients and methods:**

More than 34,000 UKA procedures carried out between 1998 and 2007 were analyzed, and we focused on over 16,000 patients younger than 65 years to determine usage and to determine differences in the revision rate. Survival analysis was used to determine outcomes of revision related to age and sex, using any reason for revision as the endpoint.

**Results:**

Both countries showed a decreasing use of UKA in recent years in terms of the proportion of knee replacements and absolute numbers undertaken per year. The 7-year cumulative risk of revision of UKA in patients younger than 65 years was similar in the two countries. Patients younger than 55 years had a statistically significantly higher cumulative risk of revision than patients aged 55 to 64 years (19% and 12%, respectively at 7 years). The risk of revision in patients less than 65 years of age was similar in both sexes.

**Interpretation:**

The results of the combined UKA data from the Australian and Swedish registries show a uniformity of outcome between countries with patients aged less than 65 having a higher rate of revision than patients who were 65 or older. Surgeons and patients should be aware of the higher risk of revision in this age group.

## Introduction

Knee replacement in patients aged less than 65 years provides a challenge due to the higher demands on knee function and a longer life expectancy.

Unicompartmental knee arthroplasty (UKA) has been an alternative treatment for knee osteoarthritis (OA) for more than 40 years, but it still remains controversial. For a successful UKA, strict indications have been suggested, including an age of more than 60 years, low activity demand, no obesity, flexion contracture of less than 5 degrees, limited angular deformity, intact anterior cruciate ligaments, no OA in the contralateral compartment, and no patellofemoral pain (Kozinn and Scott 1989). This means that UKA is a treatment suitable for a limited subgroup of patients.

In recent years, several countries have reported an increase in the use of UKA (Canadian Joint Replacement Register, The Norwegian Arthroplasty Register, National Joint Registry for England and Wales 2008, Riddle et al. 2008). Improved implant design and surgical technique have resulted in a wider range of indications (Deshmukh and Scott 2001, Pennington et al. 2003, Lisowski et al. 2004, Price et al. 2005b, Argenson and Parratte 2006, Berend et al. 2007, Mullaji et al. 2007, Emerson and Higgins 2008). Some authors have also reported good results and good prosthesis survival in young physically active OA patients (Pennington et al. 2003, Price et al. 2005a, Berend et al. 2007, Kort et al. 2007), which may have contributed to the increased use of UKA in this age group. In large national registries, however, an increased risk of revision of UKA in patients younger than 65 has been reported (Harrysson et al. 2004, Koskinen et al. 2007).

The Swedish Knee Arthroplasty Register (SKAR) was the first national arthroplasty register to record UKA, starting in 1975. In 1999, the Australian Orthopaedic Association National Joint Replacement Registry (AOANJRR) started to register knee replacements. The long history of knee replacement in Sweden in combination with large numbers in Australia enables an analysis based on complete national experience rather than on limited clinical studies that are often undertaken in highly specialized units.

Here we describe and compare the use and outcome of UKA in Australia and Sweden, with special emphasis on patients who are younger than 65 years of age.

## Patients and methods

Only UKA patients who were operated on because of OA were included. Although the SKAR provides detailed information on all UKA procedures performed between 1975 and 2007, only the 8,792 procedures that were performed between 1998 and 2007 were used in this analysis. This reflects more recent practice in UKA and corresponds to a similar period to that for which the AOANJRR could provide data. The AOANJRR provided detailed information on 25,607 UKA procedures performed between 1999 and 2007. Complete national data collection in Australia has been achieved since 2002.

Patients were initially divided into 4 age groups depending on their age at primary operation: under 55, 55–64, 65–74, and 75 or older. Our main interest was to determine and compare the outcome for UKA in patients who were less than 65 years old.

The cumulative revision rate (CRR) was calculated using any reason for revision as the endpoint. Revision was defined as a new operation in a previously operated primary UKA where 1 or more of the components was exchanged, removed, or an additional component added.

Bilateral observations were included in the data analyzed but without consideration of subject dependency, as this has been shown to be unnecessary (Robertsson and Ranstam 2003).

### Statistics

Cox proportional hazards models were used to estimate hazard ratios for revision with country, sex, and age as predictors. 4 main models were estimated: (1) revision related to country adjusted for sex and age; (2) revision related to sex and adjusted for age; (3) revision related to age dichotomized as < 65 and ≥ 65 and adjusted for sex; and (4) revision related to age dichotomized as < 55 and 55–64 and adjusted for sex.

For each model, the assumption of proportional hazards was checked. If the interaction between the predictor and the log of time was statistically significant in the standard Cox model, then a time varying model was estimated. Time points were selected based on the greatest change in hazard, weighted by a function of events. Time points were iteratively chosen until the assumption of proportionality was met; then the hazard ratios were calculated for each selected time period. If no time period is specified in our results, then the hazard ratio is over the entire follow-up period. All confidence intervals (CIs) are 95% CI.

## Results

**Figure 1.**
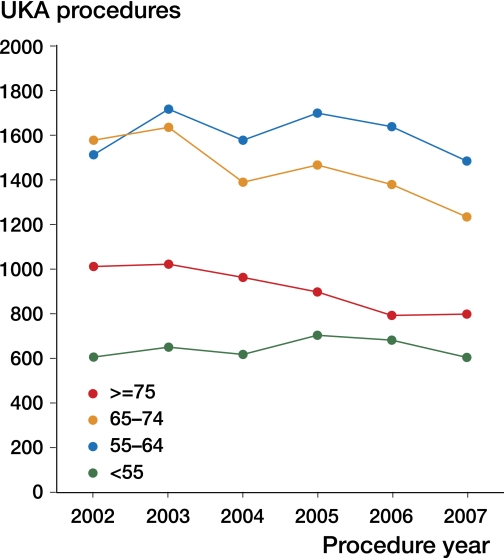
Number of UKA procedures for OA in Australia and Sweden from 2002 through 2007, by year of procedure and age.

As a proportion of the total number of knee replacements, primary UKA declined in Australia from 15% in 2002 to 9.7% in 2007 (the period of complete national data collection) and in Sweden it declined from 20% in 1998 to 7.0% in 2007 (Table 1). This was evident for all age groups (Figure 1).

**Table 1.** Number of UKAs and proportion of primary knee replacements per year in Sweden (1975–1997) and in Australia (1999–2007) for all ages

	Australia		Sweden	
Year of procedure	N	% of primary knees	N	% of primary knees
1975–1997	a	a	21,152	33
1998	a	a	1,033	20
1999	24	5.2	842	18
2000	637	12	912	15
2001	2,312	14	942	15
2002	3,860	15	908	12
2003	4,102	15	982	12
2004	3,724	12	894	9.8
2005	3,875	12	928	9.5
2006	3,625	11	903	8.6
2007	3,448	9.7	720	7.0
**^a^**Pre-registry				

In Australia UKA was more common in males (51%), except in patients younger than 55 years. In Sweden, UKA was more common in females (57%) (Table 2).

**Table 2.** The number and proportion of UKAs in Australia (1999–2007) and Sweden (1998–2007), by sex and age

Age	Australia				Sweden			
	Females		Males		Females		Males	
	N	%	N	%	N	%	N	%
<55	2,023	57	1,504	43	657	63	393	37
55–64	3,998	48	4,298	52	1,767	55	1,433	45
65–74	3,718	46	4,393	54	1,574	54	1,348	46
≥ 75	2,603	49	2,769	52	972	60	648	40
Total	12,342	49	12,964	51	4,970	57	3,822	44

In Australia, 10 UKA models accounted for 90% of UKA procedures whereas in Sweden 4 models accounted for 90% of UKA procedures (Table 3). There was a similar distribution of UKA models across all age groups.

**Table 3.** Number and percentage of UKA prostheses for OA (all ages) in Australia (1999–2007) and Sweden (1998–2007)

UKA prosthesis	N	%
Australia		
Oxford 3	8,540	34
Repicci	2,357	9.3
Unix	1,895	7.5
Preservation-Fixed	1,888	7.5
M/G	1,884	7.4
Allegretto Uni	1,652	6.5
Genesis	1,574	6.2
GRU	1,366	5.4
ZUK	798	3.2
Endo-Model Sled	776	3.1
Total	22,730	90
Sweden		
Link-Uni	3,790	43
MillerGalante-Uni	2,440	28
Oxford-Uni	1,299	15
Genesis	513	5.8
Total	8,042	91

**Figure 2.**
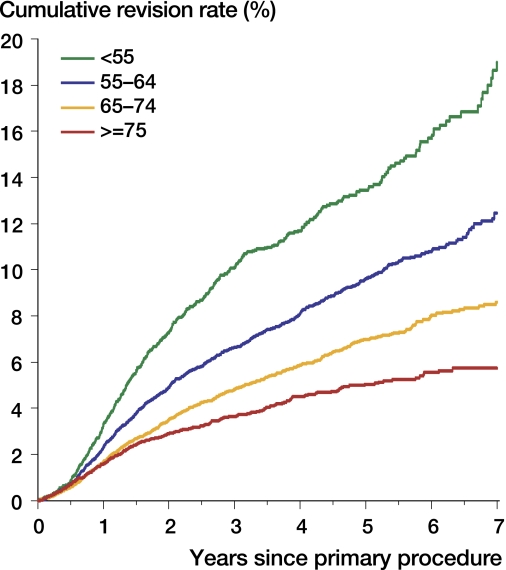
Cumulative revision rate of primary UKA for OA in Australia and Sweden, by age.

The CRR increased statistically significantly with decreasing age. At 7 years, this was 19% in patients younger than 55 years, 13% in patients aged 55–64 years, 8.6% in patients aged 65–74 years, and 5.7% in patients aged 75 years or older (Figure 2 and Table 4).

**Table 4.** 7-year cumulative revision rate (with 95% confidence interval) for primary UKA for OA, by sex and age

Age group	Males CRR (%) (95% CI)	Females CRR (%) (95% CI)	Total CRR (%) (95% CI)
< 55	19 (16–24)	19 (16–22)	19 (17–21)
55–64	12 (11–13)	13 (11–15)	13 (11–14)
65–74	7.4 (6.5–8.4)	9.7 (8.6–11)	8.6 (7.8–9.4)
≥ 75	6.0 (4.8–7.4)	5.6 (4.7–6.6)	5.7 (5.0–6.5)

Patients less than 65 years of age had a significantly higher risk of revision than patients who were 65 years or older (CRR at 7 years was 14% and 7.5%, respectively). This difference increased with time after surgery (adjusted hazard ratio (Adj HR) at 0–6 months = 1.23 (0.95–1.60), p = 0.1; Adj HR at 6 months to 1.5 year = 1.80 (1.56–2.07), p < 0.001; Adj HR at ≥ 1.5 year = 1.96 (1.74–2.21), p < 0.001). Patients less than 55 years had a higher risk of revision of primary UKA than patients aged 55–64 years for the entire follow-up period (Adj HR = 1.52 (1.36–1.70), p < 0.001). This was evident in both males and females (Figure 2 and Table 4).

**Figure 3.**
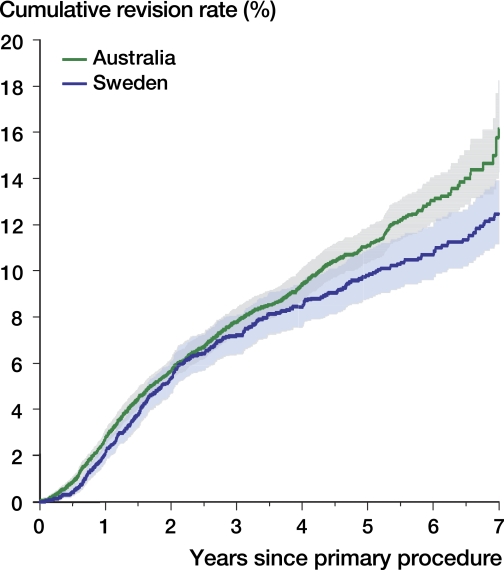
Cumulative revision rate of primary UKA in OA patients aged less than 65 years, by country. Shaded area: 95% CI.

At 7 years, the CRR in patients less than 65 years of age was somewhat higher in Australia than in Sweden, with a CRR of 16% (14–18) and 13% (11–14), respectively. However, the overall difference was not statistically significant (Adj HR = 1.13 (1.00–1.28, p = 0.05) (Figure 3).

**Figure 4.**
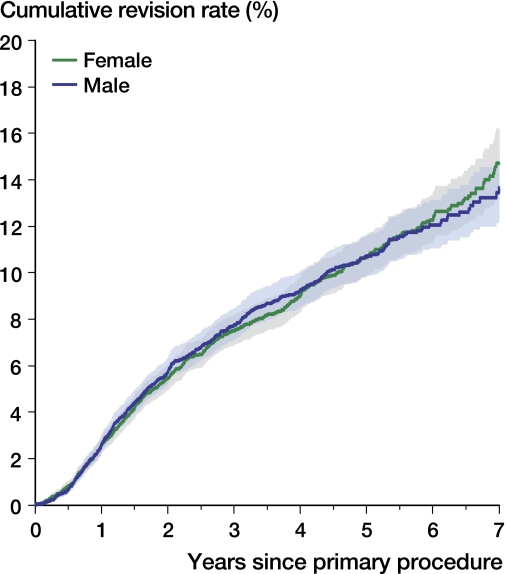
Cumulative revision rate of primary UKA in OA patients aged less than 65 years in Australia and Sweden, by sex. Shaded area: 95% CI.

The risk of revision for patients less than 65 years of age was similar in both sexes (Adj HR = 1.03 (0.92–1.15), p = 0.6) (Figure 4 and Table 4).

In Australia, 78% of the revisions of primary UKA in patients less than 65 years were to a total knee arthroplasty (TKA) and in Sweden the corresponding proportion was 91%. Loosening, progression of disease, and pain were the main reasons for revision in both Australia and Sweden. Loosening was the most common reason for revision, and it accounted for 54% of revisions in Australia and 39% in Sweden. Infection accounted for 4.3% of revisions in Australia and 2.5% in Sweden (Table 5).

**Table 5.** Main reason for revision of primary UKA in OA patients less than 65 years old in Australia and Sweden

	Australia			Sweden		
Revision diagnosis	N	% of revision	% of primary	N	% of revision	% of primary
Loosening/Lysis	506	54	4.3	142	39	3.3
Progression of disease	134	14	1.1	95	26	2.2
Pain	113	12	1.0			
Infection	40	4.3	0.3	9	2.5	0.2
Bearing/Dislocation	26	2.8	0.2			
Fracture	20	2.1	0.2	9	2.5	0.2
Wear tibial	19	2.0	0.2	13	3.6	0.3
Malalignment	17	1.8	0.1			
Instability	9	1.0	0.1	14	3.8	0.3
Dislocation	9	1.0	0.1			
Incorrect sizing	8	0.9	0.1			
Patello femoral pain	6	0.6	0.1	11	3.0	0.3
Implant breakage (femoral/tibial)	9	0.6	0.1			
Arthrofibrosis	4	0.4	0.0			
Avascular necrosis	3	0.3	0.0			
Synovitis	1	0.1	0.0			
Other	12	1.3	0.1	69	19	1.6
Unknown				4	1.1	0.1
Total revision	936	100	7.9	366	100	8.6
Total primary	11,823			4,250		

## Discussion

In recent years there has been a reported increase in the use of UKA in several countries (Canadian Joint Replacement Register; the Norwegian Arthroplasty Register, National Joint Registry for England and Wales 2008, Riddle et al. 2008). Recent studies have also shown excellent outcomes of UKA in younger patients (Pennington et al. 2003, Price et al. 2005a, Berend et al. 2007, Cartier et al. 2007, Kort et al. 2007). However, the number of patients included in these studies was small, and the studies were performed at single centers by a small number of surgeons. Combining data from the Australian and Swedish databases enabled the evaluation of usage and outcome of UKA nationally in both countries. We could also determine whether these national results supported the optimistic outcome reported for young patients in the previous studies.

SKAR registration started in 1975 when UKA was the most common type of knee arthroplasty, accounting for two-thirds of all knee procedures—the highest proportion ever reported worldwide. Since that time, the use of UKA has decreased continuously in Sweden. Similarly, the AOANJRR has experienced a major decline in the use of UKA since the collection of complete national data started in 2002. The decrease in the use of UKA may be attributed to the high risk of revision reported by both national registries over a number of years.

In Sweden, for patients of all ages with unicompartmental osteoarthritis, UKA has been a common surgical alternative. Initially, it was used most frequently in patients more than 65 years of age. In the last decade, the use of UKA has declined for patients over 65 years of age and is now more common in patients aged between 55 and 64 years.

In Australia, long-term data are not available; however, the current data show a UKA usage similar to that in Sweden (i.e. most frequent in patients between 55 and 64 years of age).

There may be several reasons for limiting the use of UKA to patients less than 65 years, the most important being less extensive surgery for less extensive disease. Shorter rehabilitation and shorter hospital stay are also benefits of UKA.

A recent study in the USA showed that in general TKA patients are more physically active, better educated, and live longer than they did several decades ago (Crowninshield et al. 2006). In combination with increased confidence in surgery and the availability of information, this may have resulted in a reconsideration of selection criteria (Pennington et al. 2003, Price et al. 2005a, Berend et al. 2007, Cartier et al. 2007, Kort et al. 2007).

Our analysis of combined data from the Australian and Swedish registries does not confirm that there are low revision rates in young patients; nor have the Finnish and Norwegian registries (The Norwegian Arthroplasty Register, Koskinen et al. 2007). In fact, patients less than 65 years of age had a higher risk of revision than patients who were 65 or older, and patients less than 55 years had a CRR of 20% at 7 years.

Australia had a higher CRR at 7 years than Sweden, but this was not statistically significant. There may be several explanations for this. There is a smaller number of types of UKA used in Sweden than in Australia, which may reduce the prosthesis-dependent variation in outcome. In addition, historically the use of UKA in Sweden has been high, and high procedure volume is known to reduce the CRR (Robertsson et al. 2001).

As a consequence of this relationship between volume and outcome, the decreasing use of UKA may have a detrimental effect on the result of this type of surgery in the future. This, in combination with the trend of operating on patients less than 65 years, may result in an increased revision burden. A potential solution is for the smaller number of UKA procedures to be undertaken by fewer surgeons who are experienced in UKA, to maintain an adequate volume of surgery and ensure optimum outcome.

It is apparent from our data that the younger the patient is when receiving a UKA, the higher is the risk of revision. It is important therefore that surgeons and younger patients should be aware of this so that they understand that UKA is unlikely to be a final surgical solution.
